# Feasibility of Using Rebound Exercise in Community‐Dwelling Adults With Neurological Disorders

**DOI:** 10.1111/nhs.70004

**Published:** 2024-11-29

**Authors:** Adaora Justina Okemuo, Yetunde Marion Dairo, Dearbhla Gallagher

**Affiliations:** ^1^ School of Health and Social Care Professions Buckinghamshire New University High Wycombe UK; ^2^ School of Human and Social Sciences‐Sports Buckinghamshire New University High Wycombe UK

**Keywords:** adults, community‐dwelling, neurological disorders, rebound exercise

## Abstract

Rebound exercise (RE) increases mobility in hospitalized adults with neurological disorders (AwND), but its feasibility in community settings remains largely unexplored. This study evaluates the practicality of implementing RE in the community, particularly for AwND. The feasibility study involved 53 community‐dwelling AwND engaging in RE sessions for 30 min, once‐ or twice‐weekly, over 12 weeks. Feasibility was assessed through recruitment rates, adherence, attrition, and participant feedback. The study measured blood pressure (BP), walking speed (WS), and physical activity level (PAL) at baseline, 6 weeks, and 12 weeks. Repeated measures ANOVA and the Friedman tests were used to test for significant differences across the time points. The study demonstrated high recruitment (70.59%) and retention (98.1%) rates, with most participants (76.9%) preferring once‐weekly sessions due to time constraints. There were no reported injuries or adverse events. Most participants were older adults (50%), females (67.3%), and retired (55.8%). Significantly lower resting BP (*p* < 0.001), higher WS (*p* < 0.001), and PAL (*p* = 0.000) were observed after 12 weeks of RE. In conclusion, RE is a feasible, safe, and acceptable intervention for supervised community‐dwelling AwND and could be a valuable tool for promoting PAL in this population.

**Summary**

- This study reveals that rebound exercise is feasible, safe, and accepted in community‐dwelling adults with neurological disorders.
- Flexible scheduling and group sessions can enhance social engagement and adherence to rebound exercise, indicating a promising strategy for community‐based neurorehabilitation.
- Rebound exercise can be a valuable tool for public health promotion efforts geared toward increasing physical activity levels.

## 1 Introduction

Rebound exercise (RE), which involves bouncing on a mini trampoline, is gaining popularity due to its therapeutic effects and numerous health benefits. It has shown promise in improving balance, strength, and mobility in various populations (Aragão et al. 2011; Márquez et al. 2010; Miklitsch et al. 2013; Okemuo, Gallagher, and Dairo 2023; Sadeghi, Ghasemi, and Karimi 2019). The body's constant movement on the unstable trampoline surface provides complex sensorimotor stimulation, leading to these benefits (Márquez et al. 2010). The gravitational forces are evenly distributed, minimizing stress on joints and bones. The trampoline's soft surface absorbs most of the body weight, reducing the impact on weight‐bearing joints (Bhattacharya et al. 1980; Burandt 2016). This makes RE ideal for individuals prone to injuries or with mobility impairments. For those less likely to engage in physically demanding activities, such as adults with neurological disorders (AwND), RE offers a low‐impact, accessible option. These individuals often experience reduced fitness and increased dependency due to balance, mobility, and strength impairments (McDonnell, Smith, and Mackintosh 2011). As a cost‐effective and time‐efficient exercise, RE has the potential to help address some of these health disparities (Bhattacharya et al. 1980; Burandt 2016).

A recent systematic review by Okemuo, Gallagher, and Dairo (2023) highlighted the potential benefits of RE on mobility, particularly when performed thrice weekly in hospital settings for AwND. However, whether these benefits can be replicated in community‐dwelling AwND remains uncertain. This is important given individuals' challenges when transitioning from a structured hospital environment to a less controlled community setting with less frequent exercise (Cowie et al. 2020; Geerligs et al. 2018; Snethen et al. 2021). Since regular exercise is vital for maintaining health, as recommended by the World Health Organization, enhancing physical activity behavior is crucial (Bull et al. 2020; WHO 2020). Exploring the feasibility of RE in a community context is essential to determine whether the positive effects seen in hospitals can be achieved outside of them.

Feasibility studies like this one are necessary for understanding how interventions like RE can be integrated into the daily lives of AwND (Eldridge et al. 2016). By assessing RE in a real‐world setting, this study aims to provide insights that can inform future interventions and strategies to improve the health and well‐being of this population. The study primarily focused on the practicality of implementing RE for AwND by evaluating participant recruitment, adherence, acceptability, safety, and logistical challenges in delivering the intervention outside a clinical environment. Additionally, it explored the potential impact of RE on physical activity behavior and physiological functions.

### 1.1 Research Questions

- Will RE be safe and feasible for AwND to use in the community?
- Will RE influence the participants' physical activity behavior and physiological function?

## 2 Methods

The study was approved by the University Research Ethics Committee (UEP, 2022Sep01) and conducted under the ethical standards of the 2013 Declaration of Helsinki (World Medical Association, 2013).

### 2.1 Research Design and Setting

This single‐group pre‐post intervention study was conducted between January and September 2023 at two Buckinghamshire New University campuses, Aylesbury and High Wycombe. “Community‐dwelling” in this study refers to participants living outside a clinical environment. The study was conducted in a research setting to closely monitor safety and adherence.

### 2.2 Sampling Technique and Sample Size

This research utilized purposive sampling techniques to recruit participants who met the inclusion criteria on the disability level as rated on the Modified Rankin Scale (mRS) and were willing to participate. Power analysis showed that 50 participants were required to get an effect size of 0.3 in walking speed at a power of 0.90 and a significance level of 0.05 (Kang 2021). While similar studies often use medium effect sizes for sample size calculations, this study had a diverse population with varying neurological conditions, which increases potential variability in outcomes. Therefore, a smaller effect size of 0.3 was chosen to account for this variability and ensure adequate statistical power.

### 2.3 Selection Criteria

Participants were community‐dwelling AwND with upper motor neuron lesions (e.g., stroke, Parkinson's disease, multiple sclerosis, and traumatic brain injury) who scored three or less on the mRS indicating mild–moderate disability (Banks and Marotta 2007), could walk for at least 2 min with or without aids, had a body weight of < 120 kg (the maximum weight capacity of the mini‐trampolines), and understood therapy instructions. An mRS score of 3 or below was utilized to ensure standardization across participants with different neurological conditions. Exclusion criteria included pregnancy and significant comorbidities such as cardiovascular disorders, severe cognitive impairment, musculoskeletal disorders, and sensory disorders affecting vision or hearing.

### 2.4 Participant Recruitment

**FIGURE 1:**
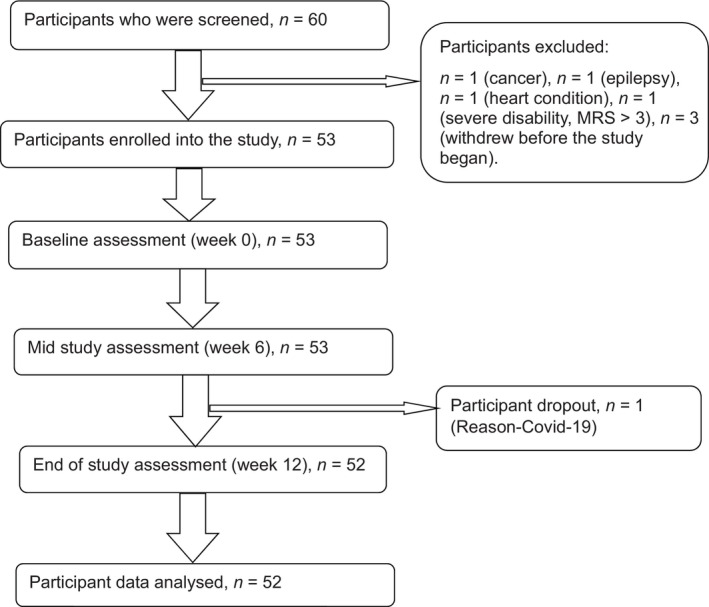
Flowchart diagram showing the participant recruitment process.

Participants were recruited from community organizations and sectors supporting AwND, including the Stroke Association, Parkinson's Disease Association, neurorehabilitation centers, and support groups. Around 85 people were approached, and those who consented signed an informed consent form after the study was explained and eligibility confirmed. Of the 60 individuals who initially expressed interest, seven were excluded based on the exclusion criteria, 53 participants were enrolled, and 52 completed the study (Figure 1).

### 2.5 Procedure for Data Collection

Enrolled participants were invited to the High Wycombe or Aylesbury campus of Buckinghamshire New University for outcome measurements and RE training. Before starting the intervention, the AHA‐ACSM Participation Screening Questionnaire was used to screen participants for cardiovascular risk factors and exercise readiness. Blood pressure (BP) measurements were taken before each RE session to ensure participants were fit to exercise.

### 2.6 Outcome Measurements

The primary outcomes were feasibility and safety, assessed through recruitment, retention rates, and adverse events. Secondary outcomes included BP, walking speed (WS), and physical activity levels (PAL), measured at baseline, 6 and 12 weeks. BP was assessed using a CAZON digital monitor (Model No: BSX556, Made in China). WS was evaluated with the validated 10‐m walk test (10MWT) (Busk et al. 2023; Cheng et al. 2020; Lindholm et al. 2018; Scivoletto et al. 2011), where participants walked 10 m as quickly as safely possible, with or without an assistive device. The time, measured using a smartphone stopwatch, was recorded twice, and the average time was used to calculate the score in meters per second by dividing the time by 10. PAL was assessed using the International Physical Activity Questionnaire (Craig et al. 2003; Roberts‐Lewis et al. 2022; Sember et al. 2020), which asked participants to recall their activities over the past week, including vigorous, moderate walking, and sitting. PAL was scored in weekly metabolic equivalent of task (MET) minutes.

### 2.7 Safety Measures and Equipment

**FIGURE 2:**
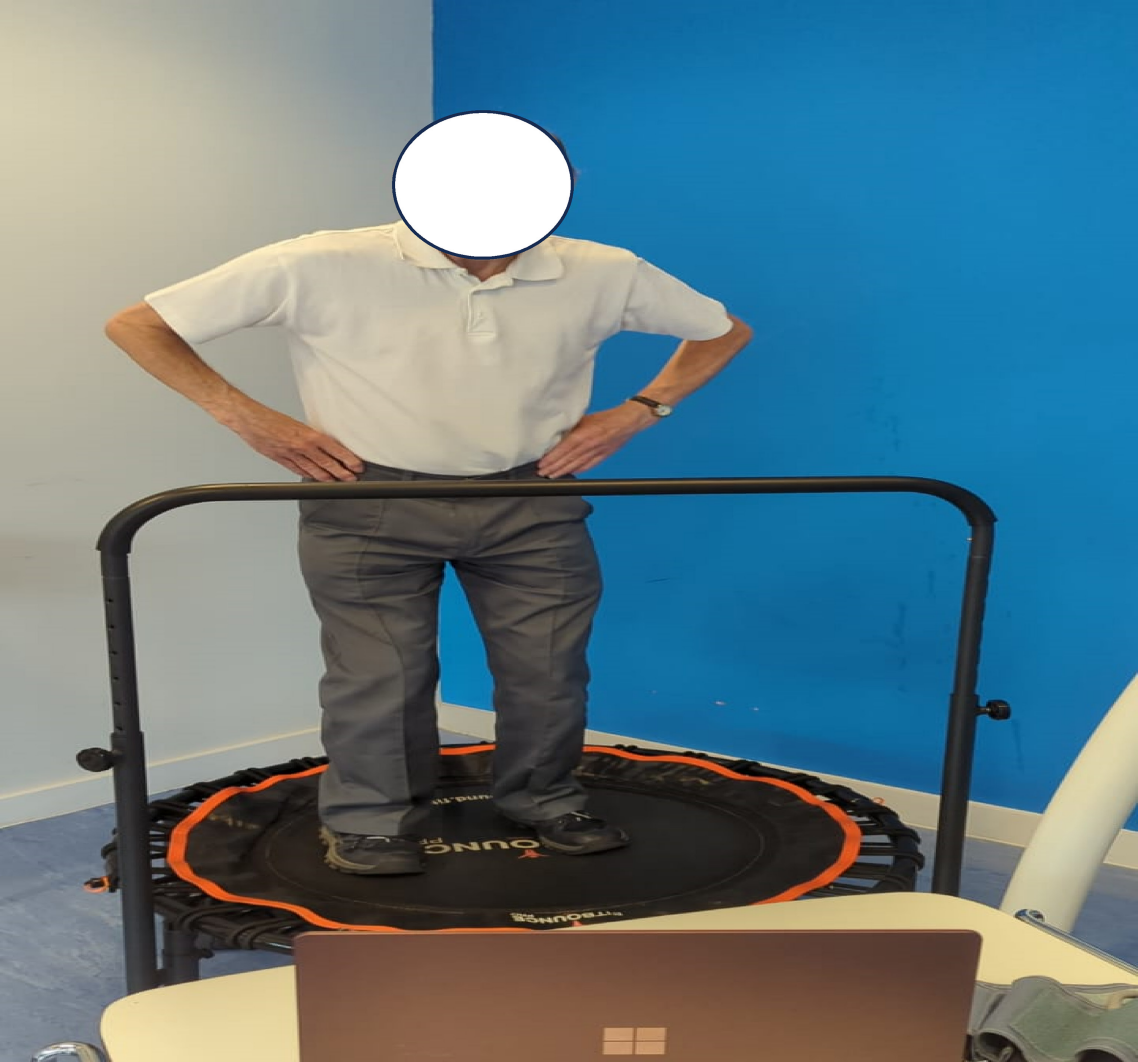
An image of a participant engaged in RE.

A single female researcher, trained in the safe and effective use of rebounders, supervised the sessions. The equipment consisted of newly purchased, sturdy mini‐trampolines (Brand: Fit Bounce Pro, Model No: 700461638780, 40 in. in diameter, with a non‐slip mat surface) fitted with stability handlebars for safety. The mini‐trampolines were placed in the corners of the room for additional support. Handgrip aids were available, but all participants had sufficient grip strength to use the handlebars without them (Figure 2). YouTube videos by RE tutor Paul Eugene were used for training. Each session began with a 5‐min warm‐up of light stretches led by the researcher. Participants then climbed onto the trampoline and followed the movements in the RE tutorial video on a laptop positioned at a comfortable viewing distance. The researcher exercised alongside the participants, offering encouragement, motivation, and assistance as needed.

Three sets of eight repetitions of the following movements were performed on the rebounder: marching‐in‐place, marching‐in‐place with arm lifts, marching in, out, front and back, jogging‐in‐place, health bounce, alternate forward kicks with bounce, bounce with alternate arm punches, weight shifts from left to right, alternate sidekicks, bouncing in and out, half jacks with unilateral arm movement, alternate hip kick and knee raise, opposite elbow to knee raise, trunk rotation from side‐to‐side, a hip shift from side‐to‐side, alternate forward foot tap, bouncing while rolling the arms, alternate side step and touch, alternate heel press, bounce boxing, modified jumping jacks, penguin high‐steppage marching, alternate heel press and toe taps, side lunges, counting down during movements, and passing a softball from one hand to another in front and behind while bouncing and free‐style bounce. Once the RE was completed, the participants were instructed to march in place on the rebounder, move slowly from side‐to‐side, and perform some five‐minute arm, leg, and trunk stretching exercises as cool‐down exercises. The duration was 30 min per session for 12 weeks.

### 2.8 Attendance and Adherence Monitoring

Participant adherence and attendance were carefully monitored throughout the study. Challenges related to scheduling were addressed by allowing flexibility in session timings, including evening and weekend options, and by permitting participants to reschedule sessions if needed. To enhance adherence, friends were allowed to attend sessions together.

### 2.9 Data Analysis

Data were summarized using descriptive statistics (frequency, percentages, medians, interquartile ranges, means, and standard deviations). A normality test was performed on all outcome variables to assess parametric testing assumptions. Repeated measures ANOVA was used for normally distributed data to test for significant differences across time points, with an alpha level set at *p* ≤ 0.05 and a 95% confidence interval. The Friedman test was applied to check for significant differences in non‐normally distributed data. Multiple regression analysis was used to test the relationship between weekly frequency and the dependent outcomes. All analyses were conducted using SPSS version 28.

## 3 Results

### 3.1 Recruitment and Retention

Approximately 85 potential participants were approached, and 60 indicated interest, resulting in a recruitment rate of 70.59%. 53 of these were eligible and enrolled. The retention rate was 98.1%, with only one participant dropping out in Week 11 due to illness.

### 3.2 Attendance Patterns

Attendance was flexible, with 40 participants (76.9%) attending once‐weekly and 12 (23.1%) attending twice‐weekly throughout the 12‐week program. Despite the flexibility, time constraints were the primary challenge, limiting most participants to once‐weekly sessions. Nevertheless, adherence was high, with all participants meeting the minimum attendance requirement of once per week.

### 3.3 Participants' Demography

Most of the participants were female (67.3%), retired (55.8%), non‐smokers (90.4%), and within the 65–74 age range (50%) (Table 1). Most of them attended once‐weekly (76.9%), had Parkinson's disease (50%), had mild disability (59.6%), and had been diagnosed between the previous 3–4 years (34.6%).

**TABLE 1:** Physical characteristics of the participants.

Index	Frequency (percentage)
Sex	
Male	17 (32.7)
Female	35 (67.3)
Marital status	
Single	18 (34.6)
Married	24 (46.2)
Divorced	7 (13.5)
Widow/widower	3 (5.8)
Age range	
35–44 years	4 (7.7)
45–54 years	6 (11.5)
55–64 years	16 (30.8)
65–74 years	26 (50)
Occupation	
Retired	29 (55.8)
Active service	23 (44.2)
Weekly frequency	
Once a week	40 (76.9)
Twice a week	12 (23.1)
Type of disease	
Stroke	14 (26.9)
Parkinson's disease	26 (50)
Huntington's disease	2 (3.8)
Multiple sclerosis	4 (7.7)
Traumatic brain injury	6 (11.6)
Duration of diagnosis	
1–2 years	9 (17.3)
3–4 years	18 (34.6)
5–6 years	6 (11.5)
7–8 years	12 (23.1)
9–10 years	6 (11.5)
Above 10 years	1 (1.9)
Severity of disability	
No disability	1 (1.9)
Mild disability	31 (59.6)
Moderate disability	20 (38.5)

### 3.4 Safety and Acceptability

No injuries or adverse events were reported during the study, indicating that the intervention was safe for community use. Participants reported enjoying the exercise, and many expressed a desire to continue the program if possible.

### 3.5 BP, Mobility and PAL

Table 2 presents the results from the repeated measures ANOVA with a Greenhouse–Geisser correction revealing statistically significant changes with large effect sizes across the time points in the SBP (*F* [1.650, 84.145] = 29.315, *p* < 0.001, *ŋ* ^2^ = 0.452), DBP (*F* [1.745, 89.006] = 32.080, *p* < 0.001, *ŋ* ^2^ = 0.485), walking speed (*F* [1.271, 64.834] = 59.611, *p* < 0.001, *ŋ* ^2^ = 0.573). For the skewed data, the Friedman test was used to test for and found significant differences in the distribution of PAL across the time points (*p* < 0.001). A post hoc pairwise comparison with the Bonferroni correction showed that these differences were significant between all the pairs (*p* < 0.001) (Table 3). After adjusting for age, disease severity, and duration of diagnosis, multiple regression analysis revealed no significant associations between weekly frequency and any of the outcomes (Table 4).

**TABLE 2:** Assessment of the distribution of outcomes across time points.

Index	Baseline mean (SD)	6 weeks mean (SD)	12 weeks mean (SD)	Test	*p*
SBP in mmHg	123.02 (7.650)	120.19 (6.356)	116.46 (5.686)	*F* = 29.315 df = 1.650	< 0.001
DBP in mmHg	78.13 (7.817)	74.63 (6.993)	71.58 (4.912)	*F* = 32.080 df = 1.745	< 0.001
Walking speed in m/s	1.4538 (0.237)	1.6850 (0.2916)	1.7890 (0.3758)	*F* = 59.611 df = 1.271	< 0.001
PAL Median [Q1–Q3] in MET min/week	2935 (2287.75–4425)	3267 (2576–4060)	3603 (3088–4329)	*X* ^2^ = 78.757 df = 2	< 0.001

*Note:* df = degree of freedom, *F* = ANOVA test statistics, *X* ^2^ = Chi‐square (Friedman test) significance level set at *p* < 0.05.

**TABLE 3:** Pairwise comparison test with the Bonferroni correction.

Index	Baseline versus 6 weeks mean (95% CI)	Baseline versus 12 weeks mean (95% CI)	6 weeks versus 12 weeks mean (95% CI)
SBP in mmHg	−2.827 (−4.170, −1.484) *p* < 0.001	−6.558 (−8.602, −4.514) *p* < 0.001	−3.731 (−5.446, −2.015) *p* < 0.001
DBP in mmHg	−3.500 (−5.113, −1.887) *p* < 0.001	−6.558 (−8.468, −4.648) *p* < 0.001	−3.058 (−4.424, −1.691) *p* < 0.001
WS in m/s	0.231 (0.172, 0.290) *p* < 0.001	0.335 (0.253, 0.418) *p* < 0.001	0.104 (0.063, 0.145) *p* < 0.001
PAL MET mins/week	358.731 (242.659, 472.802) *p* < 0.001	796.846 (567.378, 1026.314) *p* < 0.001	438.115 (260.111, 616.120) *p* < 0.001

*Note:* Adjusted significance for multiple comparisons.

**TABLE 4:** Regression analysis for weekly frequency versus dependent variables.

Dependent variable	*F*‐statistics	Degree of freedom	*p*	*R* squared	Regression coefficient (*B*)
SBP	1.234	4, 47	0.309	0.118	−0.439
DBP	0.974	4, 47	0.431	0.077	−0.851
WS	0.451	4, 47	0.771	0.037	−0.083
PAL	1.316	4, 47	0.278	0.101	−428.644

## 4 Discussion

### 4.1 Participant Demography

The study primarily included older female adult participants, most of whom experienced mild disability. This aligns with existing research showing women are more likely than men to engage in health‐related community programs, possibly due to their proactive health‐seeking behaviors and interest in physical activity (Dluhos‐Sebesto et al. 2021; Naud et al. 2019). The high number of older adults mirrors the higher prevalence of neurological disorders, such as Parkinson's, stroke, and multiple sclerosis, in this population (Dumurgier and Tzourio 2020; Huang et al. 2023). The participants' age suggests that RE may appeal to older adults as a low‐impact exercise suitable for varying mobility levels. However, while many participants had mild impairments, further research is warranted to evaluate RE's effectiveness for those with more severe disabilities.

### 4.2 Feasibility of Implementing Rebound Exercise in the Community

The study demonstrated the feasibility of implementing an RE program in a supervised community setting, with high recruitment (70.59%) and retention rates (98.1%), reflecting significant interest among AwND. This is consistent with findings from a recent study (Fricke et al. 2023). The attrition rate of just 1.89% was notably lower than the typical 30%–70% in longitudinal studies (Gustavson et al. 2012), suggesting RE is well‐tolerated and engaging, supporting participant adherence. Flexible scheduling was well‐received, with 76.9% attending once‐weekly due to time constraints, while 23.1% adhered strictly to twice‐weekly sessions, showing commitment. This preference for once‐weekly sessions aligns with findings from (Foley, Hillier, and Barnard 2010) and (Aboagye et al. 2017), indicating the practicality of flexible, lower‐frequency exercise programs for participants managing neurological disorders.

### 4.3 Safety and Acceptance

The absence of injuries or adverse events supports the safety of RE for community‐dwelling AwND, consistent with previous research (Miklitsch et al. 2013; Simonis et al. 2004). This is notable given that half of the participants had Parkinson's disease, a group prone to balance issues and falls (Fasano et al. 2017; Lima et al. 2022). Despite these risks, no safety concerns arose, highlighting the suitability of RE for this vulnerable population. It is also worth noting that the participants' baseline mean WS of 1.45 m/s suggested a relatively high functional capacity, which may have contributed to the safety. Participants also reported high enjoyment and interest in continuing, suggesting acceptance. Many were willing to buy a mini‐trampoline or join a similar program if affordable, showing potential for integrating RE into regular routines. However, further research would be needed to assess the feasibility and safety of conducting this exercise independently or in less‐supervised environments, where safety aspects may be more limited. Future programs should consider flexible scheduling and group sessions to boost participation, sustainability, and safety.

### 4.4 Challenges and Solutions

The main challenge participants reported was limited time, which led most to attend only once‐weekly. To address this, the study offered flexible scheduling, including evenings and weekends, and the option to reschedule sessions. Flexibility has been shown to improve adherence in older adults by accommodating their lifestyle needs (Fricke et al. 2023). Participants also preferred group sessions over individual ones. Allowing them to attend with friends helped alleviate concerns about exercising alone and increased motivation. This approach fostered a sense of community, enhancing motivation, mood, and adherence among older adults (Beauchamp et al. 2018; Komatsu et al. 2017).

### 4.5 Effect of RE on Physical Activity Behavior and Physiological Function

The study observed significant improvements in WS and PAL among community‐dwelling AwND after 12 weeks of RE, suggesting its potential to enhance physical activity behavior and support public health efforts. A faster WS is linked to greater independence and a lower risk of falls, particularly in older adults or those with mobility issues (Fielding et al. 2017; de Oliveira et al. 2021). RE's rhythmic and dynamic nature likely strengthens lower limb muscles and improves conditioning. The enjoyable nature of RE may also have motivated participants to stay active, aligning with research that highlights the effectiveness of enjoyable, achievable exercise programs in promoting long‐term behavioral change (Gasana et al. 2023; Lachman et al. 2018). Additionally, resting systolic and diastolic BP reductions were noted, with a 3 mmHg drop by 6 weeks and a total of 7 mmHg by the study's end. These results are consistent with findings on aerobic exercise and BP reduction (Kim and Kang 2019; Punia, Singh, and Punia 2016; Saco‐Ledo et al. 2020; Wen and Wang 2017), likely due to improved heart efficiency, reduced sympathetic nervous system activity, and enhanced vascular elasticity, leading to better blood flow and lower BP (Dimeo et al. 2012; Hegde and Solomon 2015).

### 4.6 Limitations

While this study offers valuable insights into the feasibility of RE for AwND in the community, it has limitations. The diversity in neurological disorders among participants, despite all having upper motor neuron lesions, introduces heterogeneity that may affect the generalizability of the findings, as different disorders might respond differently to the intervention. The absence of a control group also limits the ability to compare RE's specific effects against other interventions. Additionally, the 12‐week duration may not fully capture the long‐term feasibility, safety, and sustainability of RE.

### 4.7 Implications for Future Research

The findings suggest RE is a feasible and safe intervention for community‐dwelling AwND. Despite the study's primary focus on feasibility, the results suggest that even low‐frequency RE (once or twice a week) when combined with participants' usual care or activities, can potentially influence physiological and physical functions. Future studies, particularly of randomized controlled design, should explore larger sample sizes and longer durations to assess the long‐term feasibility, effectiveness, and sustainability of RE. Additionally, investigating the minimum effective dose of RE could provide insights into optimizing adherence strategies.

## 5 Conclusion

This feasibility study indicates that a community‐based RE program is practical, safe, and accepted by AwND. High recruitment and retention rates and flexible attendance options highlight the intervention's practicality in real‐world settings. The study suggests that RE may positively impact BP and physical activity behavior, aiding hypertension management and improving PAL. These findings further support the potential of RE as a complementary intervention to enhance physical and physiological outcomes in community‐dwelling AwND. Further research is needed to confirm these results in larger, more diverse populations and to explore the potential for integrating RE into standard community rehabilitation services.

### 5.1 Relevance for Clinical Practice

The study suggests that RE could be an accessible and engaging option to enhance BP and overall physical activity in this population. However, since the study was primarily designed to assess feasibility rather than efficacy, it would be premature to recommend RE for widespread clinical implementation at this stage. The results, while encouraging, need to be interpreted with caution. To ensure that RE is a safe, effective, and evidence‐based intervention for clinical practice, further rigorous studies, including randomized controlled trials, are necessary to validate these initial findings.

## Author Contributions

**Adaora Justina Okemuo:** conceptualization, methodology, software, data curation, resources, formal analysis, project administration, visualization, validation, writing – review and editing, writing – original draft, funding acquisition, investigation. **Yetunde Marion Dairo:** validation, formal analysis, supervision, resources, funding acquisition, writing – original draft, writing – review and editing. **Dearbhla Gallagher:** visualization, supervision, writing – review and editing.

## Ethics Statement

The Buckinghamshire New University Research Ethics Committee (issue no: UEP, 2022Sep01) approved the study at the start and granted ethical approval.

## Conflicts of Interest

The authors declare no conflicts of interest.

## Data Availability

The raw data from this study can be found in the OSF repository at https://osf.io/3g9dr/?view_only=d0e7eb2078ad484b8d9052014c60a47b.
